# Evaluation of the GUYS, S.T.O.N.E., and S-ReSC Scores in Predicting the Outcomes of Mini-Percutaneous Nephrolithotomy: A Prospective Study

**DOI:** 10.7759/cureus.82723

**Published:** 2025-04-21

**Authors:** Aamir Mushtaq, Saleem Wani, Feroze A Shaheen, Arif Hamid, Abdul R Khawaja, Sajad A Malik, Sajad A Para, Saqib Mehdi

**Affiliations:** 1 Urology, Sher-i-Kashmir Institute of Medical Sciences, Srinagar, IND; 2 Radiodiagnosis and Imaging, Sher-i-Kashmir Institute of Medical Sciences, Srinagar, IND

**Keywords:** guys stone score, mini-percutaneous nephrolithotomy, nephrolithometric scoring systems, s-resc score, s.t.o.n.e. score

## Abstract

Objectives

Outcomes after percutaneous nephrolithotomy (PCNL) depend on various stone-related factors that have been combined into auxiliary nephrolithometric scoring systems (NSS). Few studies have evaluated these systems in mini-percutaneous nephrolithotomy (mini-PCNL). We undertook this study to evaluate Guy’s stone score (GSS), the S.T.O.N.E. score, and the Seoul National University Renal Stone Complexity (S-ReSC) score for predicting stone-free status (SFS) and complications after mini-PCNL.

Methods

Data of patients aged 18 years and older who underwent mini-PCNL between July 2022 and June 2024 for renal stones >10 mm in largest diameter were analyzed. The clinical, perioperative, and radiological characteristics of the patients were recorded. The GSS, S.T.O.N.E., and S-ReSC scores were calculated and compared with the SFS, complications, and perioperative findings. The complete absence of stones on postoperative imaging confirmed the success of mini-PCNL. The modified Clavien-Dindo classification system was used to classify complications. Receiver operating characteristic (ROC) curve analysis was used to evaluate the performance of the scores.

Results

A total of 410 patients were included in this study. SFS was achieved in 84.4% of the cases, and complications occurred in 10.7%. All three NSSs demonstrated a statistically significant association with SFS and complications. The AUC values of the GSS, S.T.O.N.E., and S-ReSC scores for predicting SFS were 0.7172, 0.7212, and 0.7174, respectively. The predictive power for complications was inferior to that for SFS, with AUCs of 0.6112, 0.6113, and 0.6259, respectively.

Conclusion

All scores showed good predictive ability for SFS and, to a lesser extent, for complications after mini-PCNL. None of the scores outperformed other scores.

## Introduction

Percutaneous nephrolithotomy (PCNL) is the procedure of choice for large renal stones [1] and provides an excellent stone-free rate (SFR) [2]. Although it is a less invasive procedure than open renal surgery, it is still associated with significant morbidity [3-5]. Several modifications have been described to reduce the complications associated with the procedure. One such modification, mini-percutaneous nephrolithotomy (mini-PCNL), aims to reduce the size of the tract used for percutaneous access to the kidney [6,7]. Although no standardized definition exists, mini-PCNL usually implies a tract diameter of 20 French (F) or less [7]. Mini-PCNL has been seen to be associated with reduced complications in comparison to PCNL with comparable stone-free rates [8,9].

Several factors determine the outcomes, including stone-free status (SFS) and complications after PCNL, many of which have been combined into auxiliary scores. These include the Guy’s stone score (GSS), S.T.O.N.E. score, and Seoul National University Renal Stone Complexity (S-ReSC) score [10-12]. The GSS was derived through a comprehensive process that included expert opinion, a review of published data, and iterative testing [10]. The S.T.O.N.E. score is an acronym for the factors that it considers, including stone size, tract length, degree of obstruction, number of involved calyces, and essence or stone density [11]. The S-ReSC score is based on the number of renal sites occupied by stones, thereby reflecting the complexity of the stone burden [12]. These scoring systems can help counsel patients regarding their chances of achieving SFS and developing complications following PCNL. They can also act as tools to standardize the reporting of stone complexities across different studies.

While these scoring systems have been externally validated for PCNL [13-16], studies assessing the utility of these scoring systems in the context of mini-PCNL are limited [17-19]. We designed this study to assess the ability of these scoring systems to predict stone-free rates and complication rates after mini-PCNL.

## Materials and methods

After approval from the institutional ethical committee, data of 466 patients aged 18 years and above who underwent mini-PCNL between July 2022 and June 2024 for stones >10 mm in the largest diameter were prospectively collected. Of these, 38 patients did not have a computed tomography image of the kidney-ureter-bladder (CT-KUB), 10 had percutaneous nephrostomy in situ, and 8 had an ectopic or horseshoe kidney. These patients were excluded from the study, and the remaining 410 were included in the final analysis. The formula for sample size for proportions was used to calculate the sample size, with the proportion of interest calculated from the study by Labadie et al., which included 246 patients, of whom 56% achieved SFS [13]. A precision of 5% was considered, along with a 95% Confidence Interval (CI).

The Guy’s, S.T.O.N.E. score and S-ReSC score were calculated from preoperative CT-KUB images by a single radiologist as originally described by Thomas, Okhunav, and Jeon, respectively [10-12]. The operating surgeons were unaware of the stone scores. Additionally, the stone burden (SB) for each stone was calculated as 0.785 × length × width, as previously described by Smith et al. [20]. Individual stone burden was added when multiple stones were present.

Prophylactic antibiotics were administered preoperatively. The procedure was performed under general or spinal anesthesia, depending on the patient's clinical condition and the presence or absence of comorbidities. Once anesthetized, the patient was placed in the lithotomy position and draped in a sterile fashion. Under cystoscopic guidance, the ureter on the affected side was cannulated using an open-ended 5-F ureteric catheter (U-cath) over a guidewire. Once the U-cath was fixed, the patient was positioned prone and redraped. Under fluoroscopic guidance, a calyceal puncture was performed using an 18-gauge diamond-tipped puncture needle. Access was secured using a hydrophilic flexible straight-tip guidewire. The tract was dilated to 18 F. An 18-F Amplatz sheath was secured in the tract, and using a 12-F mini-nephroscope, the pelvicalyceal system was inspected, and stones were visualized. Stone fragmentation was performed using a pneumatic lithotripter. After the removal of stone fragments and confirmation of clearance visually and fluoroscopically (except in cases where obvious stone clearance could not be achieved or where the procedure needed to be abandoned), a 4.7/6-F 26 cm double J stent was placed fluoroscopically in every case, while a nephrostomy tube was placed in a few cases as deemed necessary. Operative time (OT) was defined as the time from prone positioning of the patient to insertion of a nephrostomy tube or closure of the incision. Hematocrit was measured in the immediate postoperative period, two to six hours after surgery, and changes in the hematocrit were recorded. The patients underwent X-ray KUB in the morning after surgery to check for stone clearance. In cases of any doubt, clearance was confirmed with CT-KUB 1 month after surgery. SFS was defined as the complete absence of stone fragments on the follow-up imaging. The modified Clavien grading system was used to classify complications [21]. The duration of the hospital stay was recorded from the day of surgery to the day of discharge.

Demographic parameters, stone characteristics, and intra- and postoperative details were recorded using a Microsoft Excel® spreadsheet (Microsoft Corporation, Redmond, WA, US). Demographic parameters included age, gender, body mass index (BMI), and surgical and medical history. The stone characteristics included size, SB, location, count, laterality, number of calyces involved, stone density in Hounsfield units (HU), degree of obstruction, and stone-to-skin distance. Intraoperative and postoperative details included OT, location, and number of tracts dilated, intraoperative and postoperative complications, stone-free status, fall in hematocrit, and duration of hospital stay.

Statistical analysis was done by using RStudio Version 2024.04.2+764 (RStudio Team (2020). RStudio: Integrated Development for R. RStudio, PBC, Boston, MA, US). All categorical variables were observed as numbers and percentages and were compared using the chi-squared test. Continuous data were observed as mean and standard deviation (SD) and assessed for normality of distribution using the Shapiro-Wilk test. The Mann-Whitney U test was applied as all continuous data showed a non-normal distribution. All the tests were conducted at a significance level of 5%. Receiver operating characteristic (ROC) curves were generated, and the area under the curve (AUC) was used to evaluate the ability of the GSS, S.T.O.N.E score, and S-ReSC scores in predicting SFS and complication rates. The maximum Youden index was used to define the optimal cut-off values.

## Results

A total of 410 patients who underwent mini-PCNL and met the inclusion criteria were included in this study. The mean age of the patients was 43.59 years, and 60.73% were male. SFS was achieved in 346 patients (84.4%), and complications occurred in 44 patients (10.7%). Clavien grade I complications occurred in 11 patients, Clavien grade II in 14 patients, Clavien grade III in 13 patients, and Clavien grade IV in six patients. Table 1 shows the complications observed in the study, graded according to the modified Clavien classification. One patient died during the study. Table 2 presents the demographic, stone, and perioperative characteristics of the study population.

**Table 1:** Complications graded as per the modified Clavien classification of postoperative complications n = number of patients

Modified Clavien-Dindo Complication	n	% of population
Grade I		
Fever requiring anti-pyretics	11	2.68
Grade II		
Infections requiring upgradation of antibiotics	9	2.20
Pneumonia	2	0.49
Pleural effusion	2	0.49
Ileus	1	0.24
Grade IIIa		
Peri-nephric collection	1	0.24
Pneumothorax	2	0.49
Hemopneumothorax	2	0.49
Clot retention	2	0.49
Grade IIIb		
Haematuria necessitating abandonment	1	0.24
Inability to stent	1	0.24
Pseudoaneurysm	4	0.98
Grade IVa		
Colonic Perforation	1	0.24
Grade IVb		
Urosepsis requiring inotropic support	5	1.21

**Table 2:** Demographic and perioperative characteristics of patients Parameters marked with * represent data presented as “mean ± SD”. Parameters marked with # represent data presented as “number with percentage in parentheses”. GSS: Guy’s stone score; S-ReSC: Seoul National University Renal Stone Complexity

Parameters	Values
Age (years) ^*^	43.59 ± 1.62
Male ^#^	249 (60.73)
BMI (kg/m^2^)^ *^	26.3 ± 3.1
Left side ^#^	219 (53.41)
Stone burden (mm^2^) ^*^	309.3 ± 349.16
Subcostal puncture ^#^	313 (76.34)
Number of tracts dilated ^*^	1.23 ± 0.51
Operation time (minutes) ^*^	56.03 ± 20.91
Duration of hospital stay (days) ^*^	1.82 ± 1.42
Drop in hematocrit (%) ^*^	3.4 ± 1.84
Stone-free rate ^#^	346 (84.39)
Complication rate ^#^	44 (10.73)
GSS ^#^	
Grade I	158 (38.54)
Grade II	151 (36.83)
Grade III	85 (20.73)
Grade IV	16 (3.90)
S.T.O.N.E. score ^*^	6.82 ± 1.39
S-ReSC Score ^*^	3.24 ± 1.75

When we compared the patients based on SFS, we found no difference in age, gender, BMI, laterality of stone, or location of puncture (supracostal vs. subcostal). The SB, number of tracts dilated, operative time, and duration of hospital stay were significantly higher in the group with residual stones. Patients with residual stones had a significantly higher GSS (p <0.001). The mean S.T.O.N.E scores in those with residual stones and those with complete clearance were 6.61 and 7.92, respectively, and this difference was statistically significant (p<0.001). The S-ReSC score was also significantly associated with SFS (p<0.001). Table 3 shows the comparison between patients with and without residual stones.

**Table 3:** Comparison of patients with and without stone-free status after mini-PCNL Parameters marked with * represent data presented as “mean ± SD”. Parameters marked with # represent data presented as “number with percentage in parentheses”. Statistical comparisons were performed between the stone-free group (n=44) and the not-stone-free group (n=366). A p-value of less than 0.05 (p < 0.05) was considered statistically significant. Values in bold indicate statistically significant differences. PCNL: percutaneous nephrolithotomy; GSS: Guy’s stone score; S-ReSC: Seoul National University Renal Stone Complexity

Parameter	Stone-free (n=346)	Not stone-free (n=64)	Statistical test	p
Age (years) ^*^	43.74 ± 14.71	42.77 ± 14.19	U	0.6927
Male ^#^	206 (59.53)	43 (67.18)	χ2	0.2496
BMI (kg/m^2^)^ *^	26.26 ± 3.1	26.56 ± 2.9	U	0.6457
Left side ^#^	187 (54.04)	32 (50)	χ2	0.5511
Stone burden (mm^2^) ^*^	259.08 ± 240.93	580.78 ± 620.38	U	<0.001
Subcostal puncture ^#^	269 (77.75)	44 (68.75)	χ2	0.1198
Number of tracts dilated ^*^	1.19 ± 0.49	1.45 ± 0.56	U	<0.001
Operation time (minutes) ^*^	52.99 ± 18.35	72.47 ± 25.86	U	<0.001
Duration of hospital stay (days) ^*^	1.65 ± 1.33	2.70 ± 1.56	U	<0.001
Drop in hematocrit (%) ^*^	3.33 ± 1.78	3.77 ± 2.12	U	0.2095
GSS ^#^			χ2	<0.001
Grade 1	147 (42.49)	11 (17.19)		
Grade 2	133 (38.44)	18 (28.13)		
Grade 3	61 (17.63)	24 (37.50)		
Grade 4	51 (14.74)	11 (17.19)		
S.T.O.N.E. score ^*^	6.61 ± 1.20	7.92 ± 1.80	U	<0.001
S-ReSC Score ^*^	3.01 ± 1.58	4.52 ± 2.06	U	<0.001

Age, gender, BMI, laterality, number of tracts dilated, and location of punctures were not significantly different between patients who developed complications and those who did not. Patients who developed complications had significantly higher OT, duration of hospital stay, and drop in hematocrit (p<0.001). All three scoring systems were significantly associated with the development of complications. Table 4 compares patients who developed complications with those who did not develop complications.

**Table 4:** Comparison of patients with and without complications after mini-PCNL Parameters marked with * represent data presented as “mean ± SD”. Parameters marked with # represent data presented as “number with percentage in parentheses”. Statistical comparisons were performed between the group with complications (n=64) and the group with no complications (n=346). A p-value of less than 0.05 (p < 0.05) was considered statistically significant. Values in bold indicate statistically significant differences. PCNL: percutaneous nephrolithotomy; GSS: Guy’s stone score; S-ReSC: Seoul National University Renal Stone Complexity

Parameter	Complications (n=44)	No complications (n=366)	Statistical test	p
Age (years) ^*^	40.95 ± 12.77	43.90 ± 14.81	U	0.3337
Male ^#^	31 (70.45)	218 (59.89)	χ2	0.1622
BMI (kg/m^2^)^ *^	25.88 ± 2.70	26.26 ± 3.21	U	0.2841
Left side ^#^	24 (54.55)	195 (53.28)	χ2	0.8735
Stone burden (mm^2^) ^*^	510.6 ± 589.49	285.1 ± 300.34	U	<0.001
Subcostal puncture ^#^	29 (65.91)	284 (78.02)	χ2	0.0848
Number of tracts dilated ^*^	1.32 ± 0.6	1.22 ± 0.5	U	0.3154
Operation time (minutes) ^*^	67.27 ± 23.36	54.68 ± 20.21	U	<0.001
Duration of hospital stay (days) ^*^	4.18 ± 2.58	1.53 ± 0.85	U	<0.001
Drop in Haematocrit (%) ^*^	4.55 ± 2.35	3.26 ± 1.72	U	<0.001
GSS ^#^			χ2	<0.001
Grade 1	10 (22.73)	148 (40.66)		
Grade 2	18 (40.91)	133 (36.54)		
Grade 3	12 (27.27)	73 (20.05)		
Grade 4	4 (9.09)	12 (3.30)		
S.T.O.N.E. score ^*^	7.45 ± 1.72	6.74 ± 1.33	U	0.0114
S-ReSC Score ^*^	4.00 ± 1.98	3.15 ± 1.70	U	0.0023

**Figure 1:**
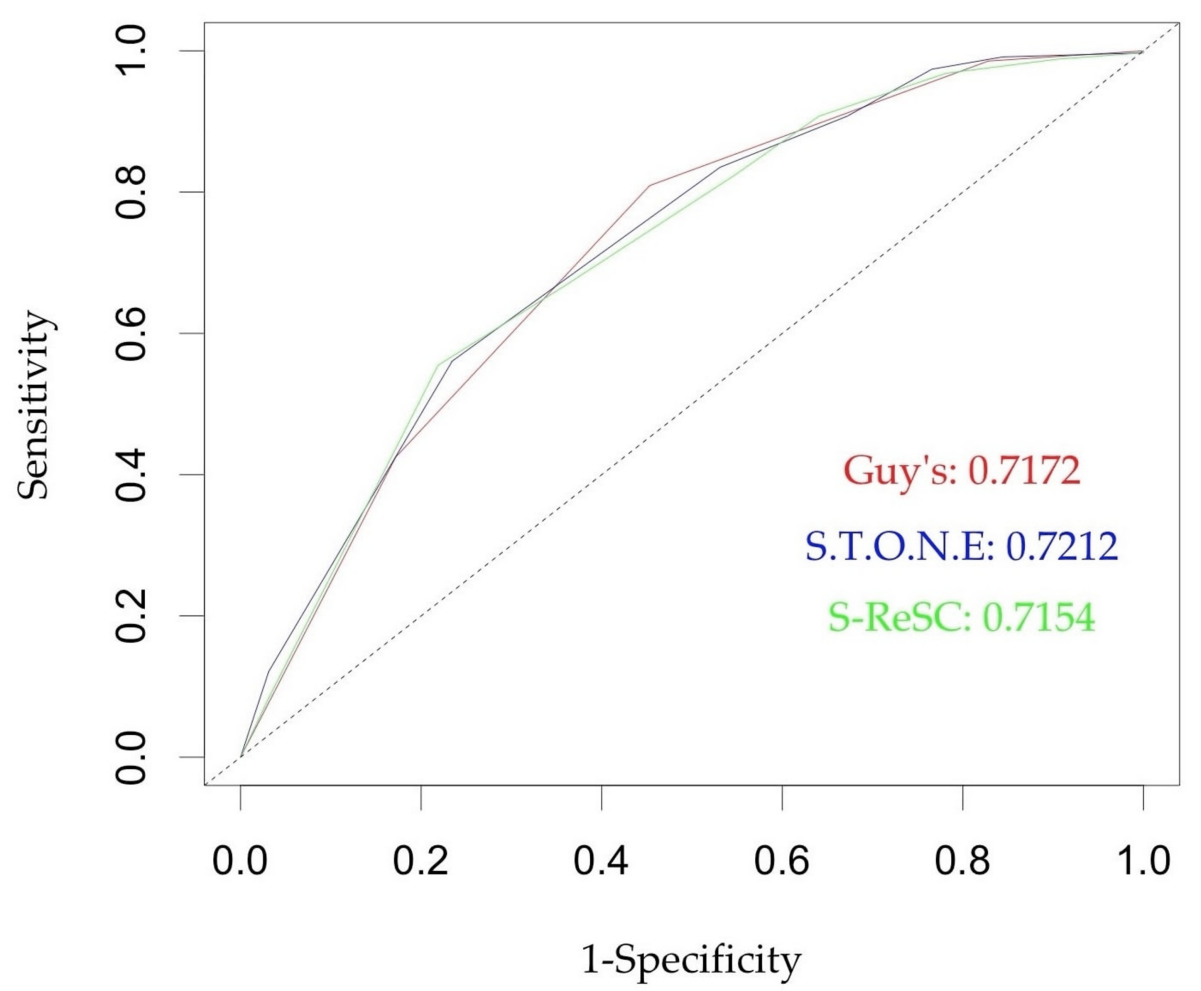
Receiver operating characteristic curves for GSS, S.T.O.N.E., and S-ReSC scores in predicting stone-free status GSS: Guy’s stone score; S-ReSC: Seoul National University Renal Stone Complexity

**Figure 2:**
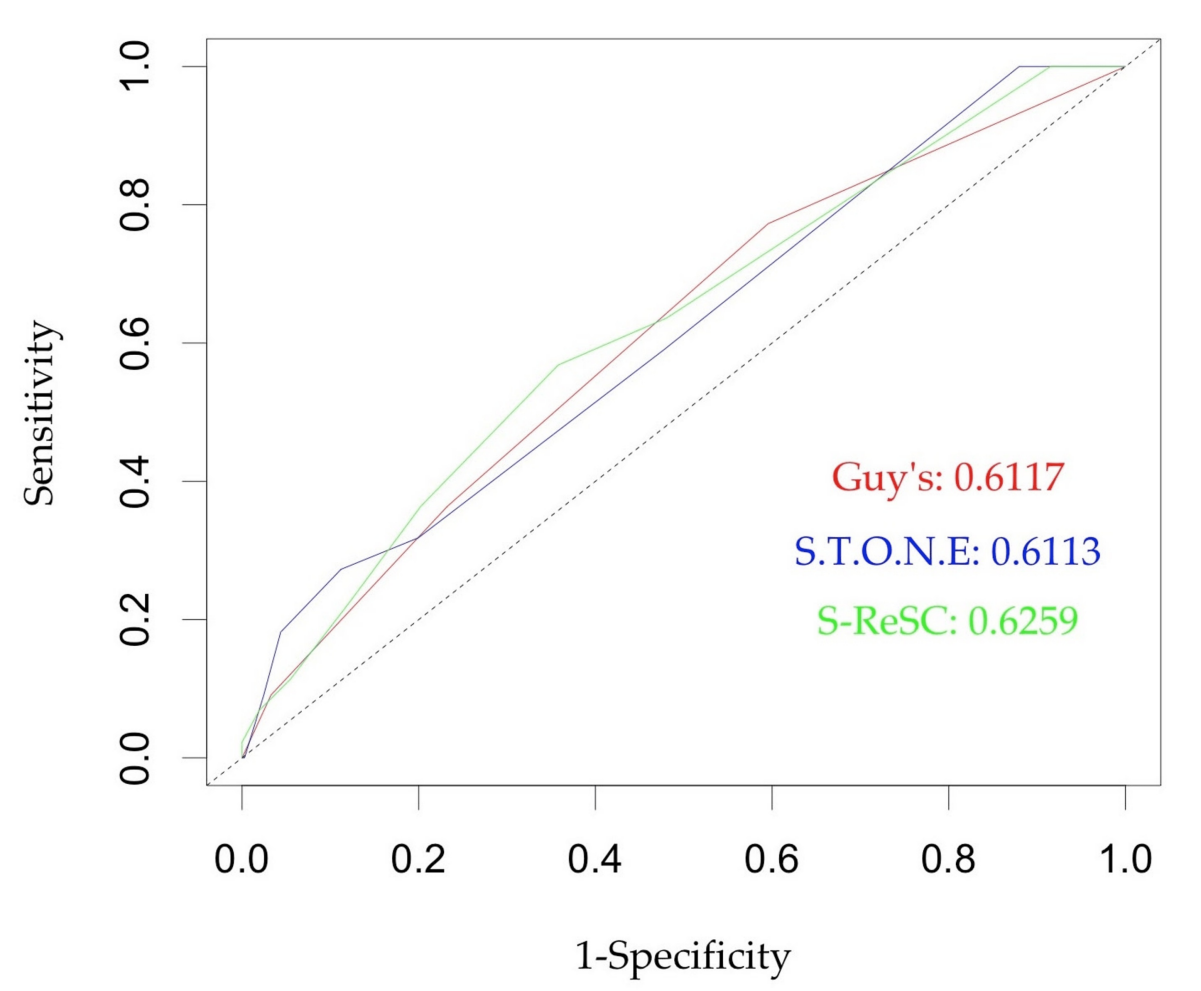
Receiver operating characteristic curves for GSS, S.T.O.N.E., and S-ReSC scores in predicting complications GSS: Guy’s stone score; S-ReSC: Seoul National University Renal Stone Complexity

Figures 1-2 show the ROC curves of the scoring systems for predicting treatment success and complications, respectively. All three scores showed good performance in predicting SFS and complications. The S.T.O.N.E. score performed the best in predicting SFS with an area under the curve (AUC) of 0.7212, while the S-ReSC performed the best in predicting complications with an AUC of 0.6259. Overall, all three scores were better at predicting SFS than complications. Tables 5-6 show the AUC, optimal cut-off, accuracy, sensitivity, and specificity of the three nomograms for predicting SFS and complications, respectively.

**Table 5:** The performance of GSS, S.T.O.N.E. score, and S-ReSC score in predicting stone-free status * 95% confidence interval in parentheses # Null hypothesis: true area = 0.5 AUC: area under the curve; GSS: Guy’s stone score; S-ReSC: Seoul National University Renal Stone Complexity

Parameter	Guy’s	S.T.O.N.E.	S-ReSC
AUC ^*^	0.7172 (0.644 – 0.790)	0.7212 (0.668 – 0.809)	0.7154 (0.644 – 0.787)
Asymptotic significance ^#^	<0.001	<0.001	<0.001
Accuracy	0.7683	0.5927	0.5902
Optimal cutoff	3	7	3
Maximum Youden Index	0.3561	0.3262	0.3362
Sensitivity	0.5469	0.7656	0.7812
Specificity	0.8092	0.5607	0.5549

**Table 6:** The performance of GSS, S.T.O.N.E. score, and S-ReSC score in predicting complications * 95% confidence interval in parenthesis # Null hypothesis: true area = 0.5 AUC: area under the curve; GSS: Guy’s stone score; S-ReSC: Seoul National University Renal Stone Complexity

Parameter	Guy’s	S.T.O.N.E.	S-ReSC
AUC ^*^	0.6112 (0.525 – 0.699)	0.6113 (0.524 – 0.699)	0.6259 (0.539 – 0.712)
Asymptotic Significance ^#^	0.012	0.013	0.004
Accuracy	0.4439	0.8220	0.6341
Optimal cutoff	2	9	4
Youden index	0.1771	0.1607	0.2103
Sensitivity	0.7727	0.2727	0.5682
Specificity	0.4044	0.8880	0.6421

## Discussion

The success of PCNL depends on several factors, including stone size, number, location, and density. Several scoring systems have been developed to incorporate these factors into a unified marker of the complexity of the procedure. However, none of these is ideal. Although these scores have shown good predictive power for SFR and mixed results for CR in standard PCNL, very few studies have assessed these scores in mini-PCNL.

GSS was originally described by Thomas et al. in 2011 [10]. It was found to independently predict SFS (p=0.01). The authors also reported good inter-rater agreement. In 2013, Okhunav et al. described the S.T.O.N.E. score using a composite of five factors [11]. They reported a significant correlation with the SFS (p=0.001). Jeong et al. described the S-ReSC score in 2103 for predicting SFS after single-tract PCNL [12].

These scores have previously been externally validated and compared to predict outcomes after standard PCNL. Ingimarsson et al. [22] and Mandal et al. [23] externally validated the GSS and reported that it significantly predicted SFS after PCNL. External validation of the S.T.O.N.E. score was performed by Okhunov et al. in a multicenter study that showed that the S.T.O.N.E. score was significantly associated with overall complication rate (p=0.0008), estimated blood loss (p=0.001), operative time (p<0.001), fluoroscopy time, and length of hospital stay(p=0.001) [24].

Labadie et al. compared the GSS and S.T.O.N.E. scores and reported that the scoring systems and stone burden were equally predictive of SFS after PCNL [13]. On ROC analysis, the AUC of the GSS and S.T.O.N.E. scores for predicting SFS were 0.634 and 0.670, respectively. The authors also reported a significant correlation with the incidence of complications. Yarimoglu et al. compared the GSS, S.T.O.N.E., and S-ReSC scores in PCNL and reported a significant correlation between the scores and SFS [14]. However, none of them could predict complications. Ozgar et al. evaluated the GSS and S.T.O.N.E. scores for predicting SFS and complications after PCNL in obese patients [15]. They reported a significant correlation between the GSS and SFS but not between the S.T.O.N.E. score and SFS. Neither of the two scores was significantly associated with complications after PCNL. Polat et al. compared the two in an elderly cohort of 147 patients [16]. The reported good predictive ability of the GSS and S.T.O.N.E score in predicting SFS after PCNL, with an AUC of 0.848 and 0.813 for the GSS and S.T.O.N.E. scores, respectively. They also reported a weaker predictive value of both scores for complications, with an AUC of 0.643 and 0.652 for the GSS and S.T.O.N.E. scores, respectively.

With mini-PCNL providing a similar SFR and a lower CR than standard PCNL, it becomes essential to look for a score that can predict the outcomes of mini-PCNL. In the mini-PCNL setting, only a few studies have attempted to evaluate these scoring systems. Ayranci et al., in 2022, were the first to investigate the ability of the GSS and S.T.O.N.E. scores to predict the outcomes of mini-PCNL [17]. The study found that neither the S.T.O.N.E. score nor the GSS was an independent predictor of SFS or complications after mini-PCNL. On ROC analysis, the AUC of the S.T.O.N.E score and GSS for predicting SFS were 0.369 and 0.414, respectively. They postulated that NSSs could not predict complications after mini-PCNL because of the low complication rates. They experienced only 13 complications during their study. Mazzon et al. compared the GSS and S.T.O.N.E. scores in a cohort of 222 patients who underwent minimally invasive PCNL [18]. They reported a good predictive value of the GSS and S.T.O.N.E. scores for predicting SFS after minimally invasive PCNL with an AUC of 0.69 and 0.62, respectively. The authors did not find a significant correlation with complications, with only a trend for GSS. However, their patient cohort was heterogeneous in that some patients underwent the procedure with a suction device and some without a suction device, which could have altered the results. Some patients also underwent the procedure using a 22-F sheath, which is outside the commonly used definition of mini-PCNL [7]. Similarly, Sigdel et al. reported their single-center experience in a cohort of 106 patients who underwent mini-PCNL [19]. Patients who were rendered stone-free had significantly lower S.T.O.N.E. scores than those with residual stones (p < 0.001). A higher S.T.O.N.E. score also had a greater risk of complications, but this was not statistically significant (p = 0.11). In our study, we found comparable AUC values for GSS, S.T.O.N.E. score, and S-ReSC score for predicting SFS (0.7172, 0.7212, and 0.7174, respectively) to those documented in the aforementioned studies. The asymptotic p-values for all three scores were <0.001. Some variations in predictive abilities could be explained by the different cutoffs used for SFS in different studies. For example, Ayranci et al. and Mazzon et al. used a cutoff of 0 mm for residual fragments [17,18]. Ozgor et al. had previously used a cut-off for residual fragments of 4 mm [15]. None of the three scores was superior to the others, with a significant overlap of the 95% confidence intervals (CI).

For predicting complications after mini-PCNL, the three scores showed an AUC of 0.6112, 0.6113, and 0.6259 for GSS, S.T.O.N.E, and S-ReSC scores, respectively, with asymptotic p-values of 0.012, 0.013, and 0.004, respectively. Overall, the predictive value of these scores for complications was inferior to the predictive value for SFS, with the lower bound of the AUC confidence intervals being <0.539. This may have rendered their predictive value inadequate. Previous studies have variably reported on the predictive ability of these scores for complications. Our positive association with complications can be attributed to the larger sample size in comparison to previous studies and, thus, a higher number of patients experiencing complications.

Considering the inconsistent literature regarding the usefulness of these scoring systems, particularly in predicting complications after mini-PCNL, a newer scoring system may be needed. Additional factors, including surgeon experience and lithotripter type, should be considered. The degree of obstruction, abnormal pelvicalyceal anatomy, and staghorn stones should be better defined. A standard definition of the SFS should also be used.

The present study had certain limitations. First, it was based on the experience of a single center. Second, our investigation focused only on the predictive value of NSSs for the success and complications of mini-PCNL. Further studies focusing on the relationship between NSS and perioperative parameters could help clarify the importance of NSS.

## Conclusions

This study represents the largest study evaluating NSSs in mini-PCNL settings. The NSSs demonstrated good predictive power for stone-free status and, to a lesser extent, for complications, as reflected by their high AUC values. This indicates that these scores are effective tools for predicting outcomes in mini-PCNL and can help guide patients preoperatively about their chances of achieving a stone-free status and developing complications. A newer scoring system incorporating factors, such as surgeon experience and type of lithotripter, as well as the standardization of definitions for SFS could further improve predictive ability.
